# Comparative Analysis of King Vision aBlade Video Laryngoscopy and Direct Laryngoscopy for Endotracheal Intubation in Paediatric Age Group: a Prospective Randomized Study

**DOI:** 10.4274/TJAR.2025.251902

**Published:** 2025-10-14

**Authors:** Mamta Harjai, Chaya Devi D, Sujeet Rai, Shilpi Misra, Tanveer Roshan Khan

**Affiliations:** 1 Dr. Ram Manohar Lohia Institute of Medical Sciences, Department of Anaesthesia and Critical Care, Lucknow, Uttar Pradesh, India; 2 Dr. Ram Manohar Lohia Institute of Medical Sciences, Department of Anaesthesiology, Lucknow, Uttar Pradesh, India; 3 Dr. Ram Manohar Lohia Institute of Medical Sciences, Department of Paediatric Surgery, Lucknow, Uttar Pradesh, India

**Keywords:** Endotracheal intubation, King Vision aBlade, direct laryngoscopy, paediatric airway management, video laryngoscopy

## Abstract

**Objective:**

Paediatric airway management is challenging due to anatomical differences, making effective endotracheal intubation crucial during surgery. While direct laryngoscopy (DL) has been the standard method, video laryngoscopy (VL) has emerged as a promising alternative. This study compared the effectiveness of King Vision aBlade non-channeled VL (KVL) with Miller/Macintosh DL for intubation in children.

**Methods:**

In this prospective, randomized, single-blinded study, 150 children aged 2-10 years undergoing elective surgery were randomly assigned to either Group DL (n = 75) or Group KVL (n = 75). Data was collected on intubation success, time, glottic view, external maneuvers, and hemodynamic parameters [heart rate (HR), systolic blood pressure (SBP), diastolic blood pressure (DBP), peripheral oxygen saturation (SpO_2_)] at various intervals.

**Results:**

The mean age of patients was similar in both groups (*P*=0.15). The DL group had a higher success rate on the first attempt (*P* < 0.001) and shorter intubation times (9.97±3.12 sec vs. 14.35±2.99 sec, *P* < 0.001) compared to KVL. Although KVL provided a better glottic view, this difference was not statistically significant (*P*=0.059). Hemodynamic parameters (SBP, DBP) were significantly higher in the DL group post-intubation (*P* < 0.05), with no significant differences in HR or SpO_2_ between groups. The DL group required more external maneuvers for intubation (*P*=0.022).

**Conclusion:**

DL showed a higher success rate, faster intubation times, and greater hemodynamic stability compared to KVL. While KVL offered better glottic views, it had longer intubation times and lower success rates. Further studies with larger sample sizes are recommended to validate these findings.

**Main Points**

• Direct laryngoscopy (DL) is faster and more successful on the first attempt compared to King Vision aBlade video laryngoscopy (KVL).

• KVL offers better glottic visualization and requires fewer external maneuvers than DL.

• KVL provides better hemodynamic stability during paediatric intubation compared to DL.

## Introduction

Airway management is a critical skill for anaesthesiologists, involving techniques such as facemask ventilation, laryngeal mask airway insertion, and endotracheal intubation using direct or video-assisted laryngoscopy.^1^ The laryngoscope, originally developed for otorhinolaryngologists, has become an essential tool in anaesthesiology for visualizing the larynx and managing the airway, particularly during endotracheal intubation. Over the past century, advancements in anaesthesia have refined the use of laryngoscopes, making them indispensable in paediatric and adult airway management.^2^

Paediatric airway management poses unique challenges due to anatomical differences, including a larger head, large tongue, cephalad larynx, and anteriorly angulated vocal cords, making laryngoscopy and intubation more difficult.^3^ Additionally, paediatric patients are more susceptible to rapid desaturation during apneic events due to lower functional residual capacity and low tidal volume.^4^ These physiological factors make securing the airway a priority, and endotracheal intubation remains the gold standard for airway management in children.^5, 6^

Direct laryngoscopy (DL), especially with the Miller blade, is the traditional method for paediatric intubation.^7^ However, recent advancements in video laryngoscopy (VL) have shown promising results, particularly in adult populations and mannequins, with VL providing better laryngeal views and improved intubation success rates. Although VL is widely used in adults, its application in paediatric airway management is still an emerging area of research.^8, 9, 10, 11, 12^

**Figure 1:**
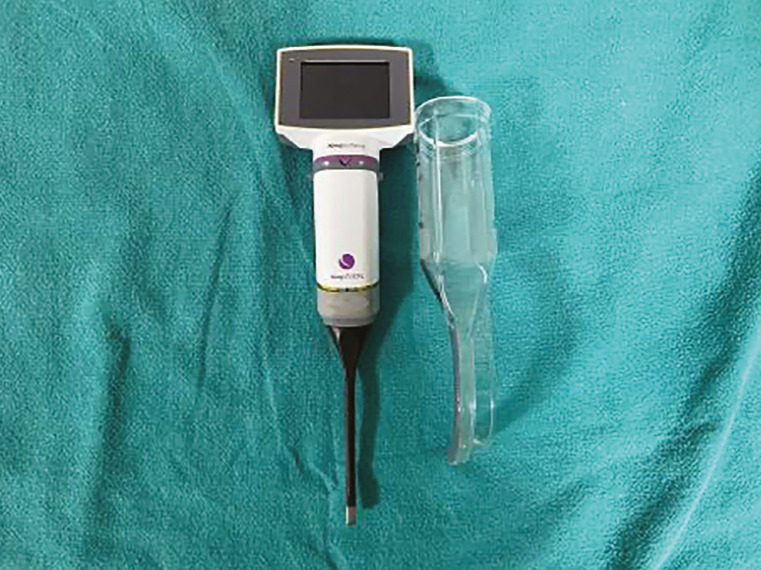
King Vision aBlade size 1.

VLs have been shown to improve glottic visualization in children, offering advantages such as superior laryngeal views, reduced force during intubation, and the ability to record and teach.^13^ The King Vision aBlade VL (KVL) (Figure 1), specifically designed for paediatric use, is a novel device that has not been extensively studied in the paediatric population aged 2 to 10 years.^14, 15^

Given the potential difficulty of intubating paediatric airways, we conducted a prospective, randomized study to compare the KVL with the Miller/Macintosh DL in children aged 2-10 years. As it is a non-channeled device with a Macintosh-like blade curvature, offering better glottic visualization and reduced lifting force. Compared to other VLs like GlideScope or C-MAC, it is portable, battery-operated, and designed to accommodate paediatric airway anatomy, making it suitable for children aged 2-10 years. We hypothesized that the time for successful tracheal intubation with the King Vision aBlade would be equivalent to that of the Miller/Macintosh blades during routine tracheal intubation in paediatric patients.

## Methods

### Study Design and Setting

This is a prospective, interventional, randomized controlled study conducted in the Department of Paediatric Surgery at Dr. Ram Manohar Lohia Institute of Medical Sciences, Lucknow, Uttar Pradesh, India. The study was performed over 18 months, with 12 months dedicated to interventions and 6 months for data analysis and thesis writing. Paediatric patients aged 2-10 years undergoing elective surgeries under general anaesthesia, which required tracheal tube intubation, were included in the study. The study was approved by the Institutional Ethical Committee of Dr. Ram Manohar Lohia Institute of Medical Sciences (approval no.: 63/19, date: 02.01.2020), and written informed consent was obtained from parents or guardians of all paediatric patients. The trial was registered with CTRI under registration number [CTRI/2020/06/025915].

### Study Participants

**Figure 2:**
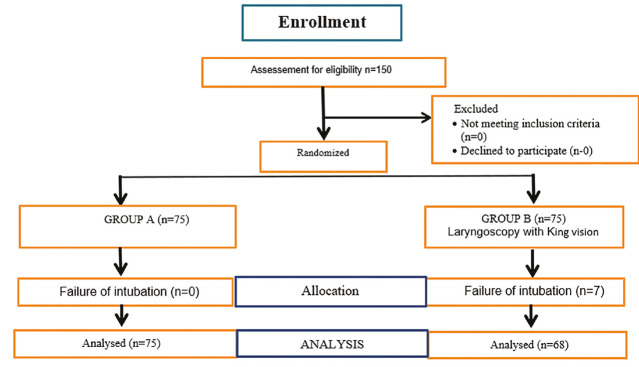
CONSORT diagram representing patient enrolment.

The inclusion criteria for the study comprised paediatric patients aged 2-10 years who were admitted for elective surgery under general anaesthesia requiring tracheal intubation, with an American Society of Anesthesiologists (ASA) physical status of I or II. Exclusion criteria included cases where parental consent for participation was not provided, patients with an ASA physical status greater than II, those with active urinary tract infections, and patients with congenital anomalies or an anticipated difficult airway. Additionally, any patient in whom tracheal intubation could not be successfully achieved after three attempts using either laryngoscopy method was also excluded from the study (Figure 2).

### Sample Size Calculation

Based on the previous study Jagannathan et al.,^15^ the difference in the mean duration of time for tracheal tube entry (from the device into device out (sec) (μ1-μ2) was in the Miller group (12.3) and King Vision group (18.2) and the average population variance (σ2) in 11.9 (Jagannathan et al.,^15^). The sample size (n) = 2 (Zα/_2_ + Z_[1-β]_)^2^ × σ^2^/(μ1-μ2)^2^, assuming 0.05 level significance (Z_α/2 _= 1.96), and 80% power (Z _[1-β]_)=0.84) is 63.79 in each group. Considering any dropouts, we will enroll 75 patients in each group:

n = [2 (Zα/_2_ + Z_[1-β]_)^2^ × σ^2^]/(μ1-μ2)^2^

n = [2 (1.96 + 0.84)^2^ × 11.9^2^]/(18.2-12.3)^2^

n = 150

### Study Groups

Patients were randomly divided into two groups:

●** Group DL:** Patients intubated using the Miller or Macintosh laryngoscope.

●** Group KVL:** Patients intubated using the King Vision aBlade non-channeled VL.

### Hypothesis

Endotracheal intubation with King Vision aBlade non-channeled VL is equivalent to intubation with the DL.

### Randomization, Allocation Concealment, and Blinding

### Randomization

**Sequence generation:** A computer-generated random number table was used for randomization into the two study groups. Block randomization with a variable block design was employed to ensure balanced allocation between the groups.

**Allocation concealment:** Allocation was concealed using sequentially numbered opaque envelopes. Each patient who met the inclusion and exclusion criteria and provided consent for participation was assigned to one of the two groups after their name was entered on the cover of a sequentially numbered envelope. The treatment groups were encoded as Group 1 (DL) and Group 2 (KVL), with the code kept in a sealed envelope in a secure location, only to be opened after the data analysis was complete.

**Implementation:** The generation of random numbers and the preparation of sealed envelopes were done by a statistician who was not involved in the study. The code for the groups was also kept with the statistician in a sealed envelope until the principal investigator had finished the data analysis.

### Blinding

This study was conducted as a single-blind trial, where the outcome assessor was blinded to group allocation. The study groups (DL and KVL) were randomly encoded as Group 1 and Group 2, and the code was hidden from both the patients and the data analyst until the study’s completion. However, the anaesthesiologist performing the procedure was aware of the group assignment due to the inherent differences in anatomical positioning required for each intubation technique.

### Intervention

### Group DL

In Group DL, patients were intubated using either a Miller or Macintosh laryngoscope, with blade sizes 1 or 2 selected based on the patient’s anatomy. The Cormack-Lehane grade of the glottic view was recorded during the procedure to assess the visibility of the laryngeal structures. Additionally, the time required for intubation was measured, defined as the interval from the entry of the laryngoscope blade into the mouth to the detection of the first end-tidal CO_2_.

### Group KVL

In Group KVL, patients were intubated using the King Vision aBlade non-channeled VL, with blade size 2. As in Group DL, the Cormack-Lehane grade of the glottic view was recorded to assess the visual clarity of the laryngeal aperture. The time to intubation was similarly documented, using the same criteria as in Group DL, from the blade’s entry to the detection of the first end-tidal CO_2_.

All patients underwent a detailed preoperative airway evaluation, including body mass index, ASA grading, and Modified Mallampati Grading to predict intubation difficulty. The operating room was prepared with all necessary equipment, including laryngoscopes, endotracheal tubes, and emergency drugs, to handle any airway complications. After securing an intravenous line, patients were pre-oxygenated with 100% oxygen for three minutes.

Anaesthesia was induced using sevoflurane (3-6%), fentanyl (2 μg kg^-1^), and a muscle relaxant, either atracurium (0.5 mg kg^-1^) or cisatracurium (0.1-0.2 mg kg^-1^). Hemodynamic parameters, such as heart rate (HR), systolic and diastolic blood pressure, and oxygen saturation, were monitored and recorded at various intervals, including pre-induction, immediately after intubation, and at 1, 3, and 5 minutes post-intubation. The success of intubation, the number of attempts, and the use of any external maneuvers, such as the BURP maneuver, were documented. In cases of failed intubation, corrective actions were taken and recorded. All intubations were performed by a senior anaesthesiology resident in the final year of training (3^rd^ year), under supervision.

Procedures were conducted in accordance with the Helsinki Declaration-2013.

### Statistical Analysis

Statistical analysis was performed using SPSS version 21.0 (Chicago, Inc., USA). Categorical data were analyzed using the chi-square test, while continuous variables were compared using a Student’s t-test. For comparisons involving more than two variables, one-way analysis of variance (ANOVA) was employed. The level of statistical significance was set at *P *< 0.05. Mean and standard deviation (SD) were calculated for continuous variables, providing a measure of central tendency and variability, respectively. The chi-square test was utilized to evaluate differences between categorical data, ensuring an assessment of the association between variables. The Student’s t-test was used to compare the means of two groups, while the one-way ANOVA test was applied to analyze differences among groups with more than two variables. A *P *value of less than 0.05 was considered statistically significant throughout the study.

## Results

This prospective, single blinded, randomised control study was conducted in 150 paediatric patients, undergoing elective surgery under general anaesthesia, to do a comparative analysis of KVL and DL for endotracheal intubation in paediatric population 2-10 years.

The mean age of patients in the DL group was 6.01±2.71 years, while in the KVL group, it was 5.42±2.20 years. This difference is not statistically significant (*P*=0.15), indicating that the age distribution between the two groups is comparable. There was no significant difference in sex distribution (*P*=0.47) or age (*P*=0.15) between the groups. However, the DL group had marginally taller patients, with a borderline significant *P *value of 0.05. The DL group also had significantly heavier patients than the KVL group (*P*=0.01). Both groups consisted entirely of patients with ASA status I, indicating no systemic disease, with no variation in ASA status between them (Table 1).

The DL group had significantly more successful intubations on the first attempt compared to the KVL group (*P *< 0.001). Additionally, the time for intubation was significantly shorter in the DL group (9.97±3.12 seconds) than in the KVL group (14.35±2.99 seconds, *P *< 0.001). Although the Cormack-Lehane glottic view was better in the KVL group, the difference was not statistically significant (*P*=0.059). The need for external maneuvers (e.g., BURP) was significantly higher in the DL group (*P*=0.022). No blade changes were required, and all intubations were performed by a single operator in both groups (Table 2). In the DL group, 64% (48/75) of patients were intubated using the Miller blade and 36% with the Macintosh blade, based on anatomical suitability.

**Figure 3:**
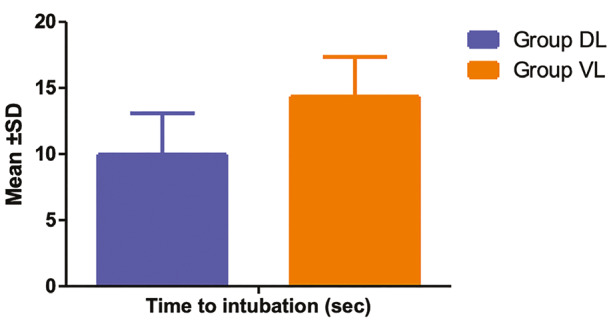
Bar chart shows the comparisons of mean time to intubation (sec) in between group DL and group VL. DL, direct laryngoscopy; VL, video laryngoscopy; SD, standard deviation.

Figure 3 compares the mean time to intubation between the DL and KVL (King Vision Laryngoscopy) groups. The mean time to intubation in the DL group was 9.97±3.12 seconds, whereas in the KVL group, it was significantly higher at 14.35±2.99 seconds. The difference between the two groups was statistically significant (t = -8.54, *P *< 0.001), indicating that intubation with the King Vision laryngoscope took longer than with the direct laryngoscope. A preformed stylet was used in all patients in the KVL group to aid in tube navigation due to the curvature of the blade. In the DL group, a stylet was used in 18.6% of cases where difficulty was encountered during the first attempt.

The hemodynamic parameters, including HR, systolic blood pressure (SBP), diastolic blood pressure (DBP), and oxygen saturation (SpO_2_), were assessed at three different time intervals: before intubation, 1 minute after intubation, and 3 minutes after intubation. Significant differences were found in SBP and DBP between the DL and VL groups across all time points, with the DL group consistently showing higher values (*P *< 0.05). Specifically, SBP and DBP were significantly higher in the DL group both after 1 and 3 minutes of intubation compared to the VL group. However, no statistically significant differences were observed in HR or SpO_2_ levels at any of the three time intervals between the two groups (*P *> 0.05) (Table 3).

The success rate for intubation within 10 seconds, using only one attempt and without external maneuvers, between the DL and VL groups was significantly higher in the DL group (54.67%) compared to the VL group (13.24%), with a *P *value of <0.001, indicating a statistically significant difference between the two groups.

## Discussion

The main finding in this study is that, the mean time required for tracheal intubation with KVL was found considerably longer (>4 sec) as compared to group DL in 2-10 yrs, of paediatric population. This finding was statistically significant. Although the percentage of success rate for intubation was less with in KVL compared to in DL group which was statistically significantly. KVL provides a better laryngoscopic view of glottis with CL grading in most of paediatric patients than that of DL.

Based on our results KVL time to intubation is longer as compared to DL n significant. The result is in concordance with similar study with KVL in paediatric age group of <2 years the time to intubation was significantly longer in VL group^7^ and also the intubation time with glidoscope was longer compared to conventional DL.^16^ Since this finding is in concordance with results of many studies as mentioned above, we can conclude that with VL time to intubation is longer as compared to DL probably because of requirement of additional hand-eye co-ordination for tube manipulation and difficulty in manoeuvring of tracheal tube through the vocal cords. The longer intubation time and lower first-attempt success with KVL may be attributed to the need for hand-eye coordination, less familiarity with the device, and the lack of a channeled blade making tube advancement through the glottis more difficult.

Use of King Vision VL was found associated with better glottic visualisation on laryngoscopy as the glottic view for C-L grade 1, 2, and 3 in our Study. We discovered that the KVL provided a superior view of the glottis than the other groups, although there was no statistically significant difference. These results are similar to other studies with Stortz VL,^17^ Glidoscope,^16^ Berci-Kaplan VL.^18^ Therefore with VL there is improved vision of larynx. External manipulation was necessary for manipulation of larynx for glottic visualisation during intubation in both groups requirement of BURP was more in DL group compared to KVL group, which was statistically significant among both the group similar to other studies.^16, 17, 18, 19, 20^ We conclude that better visualization of larynx with VL results in less use of external manoeuvres.

### Study Limitations

Our study had several limitations. First, the sample size may be considered small, and a larger study is warranted to confirm the findings in this paediatric age group (2-10 years). Second, blinding was not feasible as it was impractical to blind the operator to the device being used. This could introduce bias in favor of the standard device (DL) when comparing the performance of the new device (King Vision aBlade). Third, while the intubations were performed by anaesthesiologists experienced with videolaryngoscopy using devices like True View and Airtraq, their experience with the King Vision aBlade in this paediatric population was limited. Although the blade of the KVL has a shape similar to the Macintosh blade, the intubation technique differs, and the learning curve for advancing the endotracheal tube under various VLs cannot be ignored. This is reflected in our study, as the technique requires adequate training and experience. Additionally, the study only included children with normal airways, so the results cannot be extrapolated to those with abnormal airways. Lastly, we exclusively studied the non-channeled King Vision laryngoscope with a size 2 blade (although younger or smaller patients might benefit from a size 1 blade), and the findings cannot be applied to channeled King Vision blades or other VLs with a similar morphology.

## Conclusion

Our hypothesis that KVL is equivalent to conventional DL was not achieved because time to intubation was more in KVL (>4 secs) as compared to DL in 2-10 yrs, of paediatric population. More studies with larger sample size are warranted in future to confirm such findings. KVL provides a better laryngoscopic view of glottis with CL grading in most of paediatric patients than that of DL. Although this study demonstrated that DL outperformed KVL in several key areas of paediatric intubation. DL showed a significantly higher success rate on the first attempt, with faster intubation times compared to KVL. Although KVL provided a better glottic view, the difference was not statistically significant. Hemodynamic parameters, specifically SBP and DBP, were significantly higher in the DL group post-intubation. Additionally, the success rate for intubation within 10 seconds, without external maneuvers, was significantly higher in the DL group. These findings suggest that while KVL offers some advantages in visualization, DL remains more efficient for paediatric intubation in terms of time and ease of procedure.

## Ethics

**Ethics Committee Approval:** The study was approved by the Institutional Ethical Committee of Dr. Ram Manohar Lohia Institute of Medical Sciences (approval no.: 63/19, date: 02.01.2020).

**Informed Consent:** Written informed consent was obtained from parents or guardians of all paediatric patients.

## Figures and Tables

**Table 1. Patient Characteristics in Both Groups:** 

**Variables**		**DL (n = 75)**	**KVL (n = 68)**	***P *value**
Sex	Female	15	17	0.47
	Male	60	51	
Age (years) Mean ± SD		6.01± 2.71	5.42±2.20	0.15
Height (cm) Mean ± SD		120.39±10.3	116.80±12.02	0.05
Weight (kg) Mean ± SD		22.69±5.78	20.48±4.23	0.01
ASA status	I	75	68	NA
	II	0	0	
	III	0	0	

DL, direct laryngoscopy; KVL, King Vision aBlade video laryngoscopy; SD, standard deviation; ASA, American Society of Anesthesiologists

**Table 2. Comparative Data During Tracheal Intubation Across Both Groups:** 

-		**DL (n = 75)**	**KVL (n = 68)**	***P *value**
Intubation attempts (n)	1	64	40	<0.001*
	2	11	28	
Time for intubation (s) (Mean ± SD)		9.97±3.12	14.35±2.99	<0.001*
Glottic view (n) Cormack-Lehane	1	25	36	0.059
	2	48	31	
	3	2	1	
	4	0	0	
External manoeuvre	BURP	31	15	0.022*
	None	44	53	
Change of blade		0	0	-
Number of operators	1	75	68	-

DL, direct laryngoscopy; KVL, King Vision aBlade video laryngoscopy; SD, standard deviation

**Table 3. Comparisons of Hemodynamic Parameters in Between Group DL and Group VL at Various Time Intervals (Mean±SD):** 

**Variables**		**DL (n = 75)**	**VL (n = 68)**	***P *value**
Before intubation	HR (Beat/min)	111.87±15.16	115.90±12.59	0.088
	SBP (mmHg)	105.05±9.34	101.76±9.11	0.035
	DBP (mmHg)	65.65±7.91	63.68±8.77	0.159
	SpO_2_ (%)	100.00±0.00	100.00±0.00	-
After 1 min intubation	HR (Beat min)	125.13±13.89	126.10±13.97	0.678
	SBP (mmHg)	113.95±9.11	109.24±9.79	0.003
	DBP (mmHg)	73.21±8.72	69.46±8.28	0.009
	SpO_2_ (%)	100.00±0.00	100.00±0.00	-
After 3 min of intubation	HR (Beat min)	121.72±14.06	120.85±15.13	0.723
	SBP (mmHg)	110.64±9.52	106.43±10.98	0.015
	DBP (mmHg)	71.52±9.66	65.97±11.63	0.002
	SpO_2_ (%)	100.00±0.00	100.00±0.00	-

DL, direct laryngoscopy; KVL, King Vision aBlade video laryngoscopy; VL, video laryngoscopy; SD, standard deviation; HR, heart rate; SBP, systolic blood pressure; DBP, diastolic blood pressure; SpO_2_, peripheral oxygen saturation

## References

[1] Harless J, Ramaiah R, Bhananker SM (2014). Pediatric airway management. Int J Crit Illn Inj Sci.

[2] Goldmann K (2006). Recent developments in airway management of the paediatric patient.. Curr Opin Anaesthesiol.

[3] Fiadjoe JE, Nishisaki A, Jagannathan N (2016). Airway management complications in children with difficult tracheal intubation from the Pediatric Difficult Intubation (PeDI) registry: a prospective cohort analysis.. Lancet Respir Med.

[4] Jagannathan N, Sequera-Ramos L, Sohn L (2015). Randomized comparison of experts and trainees with nasal and oral fibreoptic intubation in children less than 2 yr of age.. Br J Anaesth.

[5] Passi Y, Sathyamoorthy M, Lerman J, Heard C, Marino M (2014). Comparison of the laryngoscopy views with the size 1 Miller and Macintosh laryngoscope blades lifting the epiglottis or the base of the tongue in infants and children< 2 yr of age.. Br J Anaesth.

[6] Aziz M, Dillman D, Kirsch JR, Brambrink A (2009). Video laryngoscopy with the macintosh video laryngoscope in simulated prehospital scenarios by paramedic students.. Prehosp Emerg Care.

[7] Byhahn C, Iber T, Zacharowski K, Weber CF, Ruesseler M, Schalk R, Meininger D (2010). Tracheal intubation using the mobile C-MAC video laryngoscope or direct laryngoscopy for patients with a simulated difficult airway.. Minerva Anestesiol.

[8] Jungbauer A, Schumann M, Brunkhorst V, Börgers A, Groeben H (2009). Expected difficult tracheal intubation: a prospective comparison of direct laryngoscopy and video laryngoscopy in 200 patients.. Br J Anaesth.

[9] Low D, Healy D, Rasburn N (2008). The use of the BERCI DCI® video laryngoscope for teaching novices direct laryngoscopy and tracheal intubation.. Anaesthesia.

[10] McElwain J, Malik MA, Harte BH, Flynn NM, Laffey JG (2010). Comparison of the C‐MAC® videolaryngoscope with the Macintosh, Glidescope®, and Airtraq® laryngoscopes in easy and difficult laryngoscopy scenarios in manikins.. Anaesthesia.

[11] Ng I, Sim XL, Williams D, Segal R (2011). A randomised controlled trial comparing the McGrath® videolaryngoscope with the straight blade laryngoscope when used in adult patients with potential difficult airways.. Anaesthesia.

[12] Vlatten A, Aucoin S, Gray A, Soder C (2009). Difficult airway management with the STORZ video laryngoscope in a child with Robin Sequence.. Pediatr Anesth.

[13] Riveros R, Sung W, Sessler DI (2013). Comparison of the Truview PCDTM and the GlideScope (®) video laryngoscopes with direct laryngoscopy in pediatric patients: a randomized trial.. Can J Anaesth.

[14] Kim JT, Na HS, Bae JY (2008). GlideScope® video laryngoscope: a randomized clinical trial in 203 paediatric patients.. Br J Anaesth.

[15] Jagannathan N, Hajduk J, Sohn L (2017). Randomized equivalence trial of the King Vision aBlade videolaryngoscope with the Miller direct laryngoscope for routine tracheal intubation in children< 2 yr of age.. Br J Anaesth.

[16] Das B, Samanta A, Mitra S, Jamil SN (2017). Comparative evaluation of Airtraq™ optical Laryngoscope and Miller’s blade in paediatric patients undergoing elective surgery requiring tracheal intubation: A randomized, controlled trial.. Indian J Anaesth.

[17] Xu X, Ma H, Zhang Y (2024). Efficacy of bougie first approach for endotracheal intubation with video laryngoscopy during continuous chest compression: a randomized crossover manikin trial.. BMC Anesthesiol.

[18] von Hellmann R, Fuhr N, Maia IW (2024). Effect of bougie use on first-attempt success in tracheal intubations: a systematic review and meta-analysis.. Ann Emerg Med.

[19] Vlatten A, Aucoin S, Litz S, Macmanus B, Soder C (2009). A comparison of the STORZ video laryngoscope and standard direct laryngoscopy for intubation in the Pediatric airway–a randomized clinical trial.. Paediatr Anaesth.

[20] Weiss M, Hartmann K, Fischer J, Gerber AC (2001). Video-intuboscopic assistance is a useful aid to tracheal intubation in pediatric patients.. Can J Anesth.

